# Supplementary material for: Patient-derived colon epithelial organoids reveal lipid-related metabolic dysfunction in pediatric ulcerative colitis
Source: Nat Commun. 2025 Dec 10;16:11026. doi: 10.1038/s41467-025-65988-2 (PMC12695892; doi:10.1038/s41467-025-65988-2)
Supplement: Supplementary file 1 — Supplementary Information [file 41467_2025_65988_MOESM1_ESM.pdf]

Supplementary Information for

# Patient-derived colon epithelial organoids reveal lipid-related metabolic dysfunction in pediatric ulcerative colitis

Babajide A. Ojo et al.

## Supplementary Figures

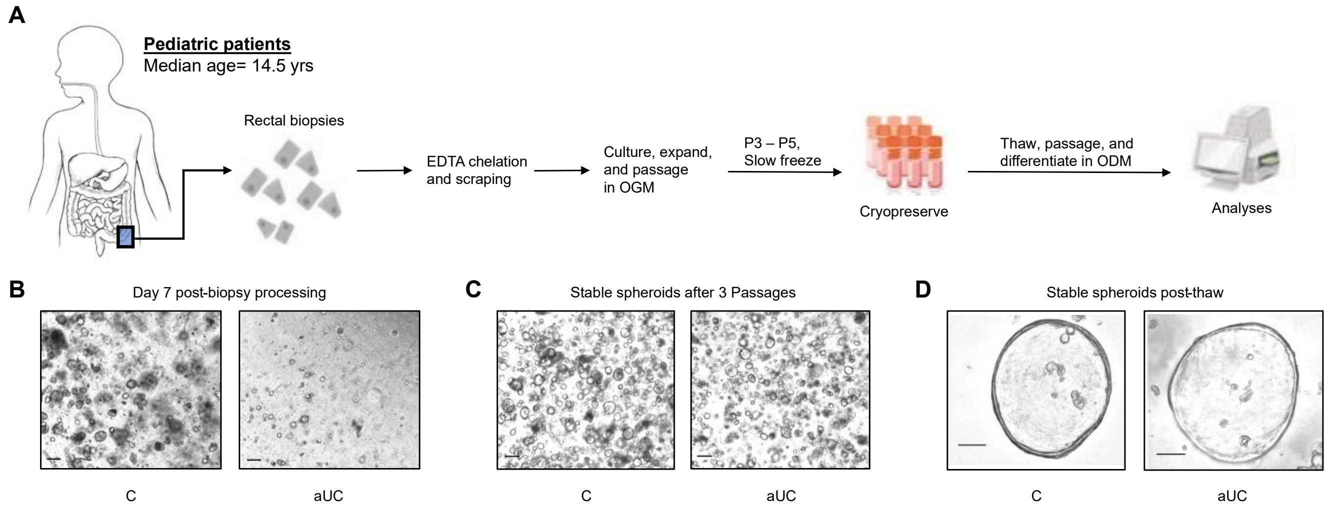


**Supplementary Fig. 1. Colon Epithelial Organoid Generation**

(**A**) Schematic of epithelial organoid derivation and maintenance from pediatric rectal biopsies. See Methods for details. The demographics of donors are provided in Supplementary Table 1. Some icons were obtained from Biorender, Ojo, B. (2025) https://BioRender.com/nwwqemf. (**B**) Phase-contrast images of C and aUC spheroids after the first 7 days of biopsy derivation in OGM show that the development of aUC spheroids from biopsies is slower than the C spheroids. Scale bar, 200 µm. (**C**) Phase contrast images of C and aUC spheroids in culture after 3 passages, showing that after initial slow growth rates, aUC spheroids become more stable like C spheroids. Scale bar, 200 µm. (**D**) High magnification images of C and aUC spheroids, showing expected

morphology with large lumens after freeze-thaw protocol. Scale bar, 50 µm. OGM, Organoid Growth Medium; ODM, Organoid Differentiation Medium

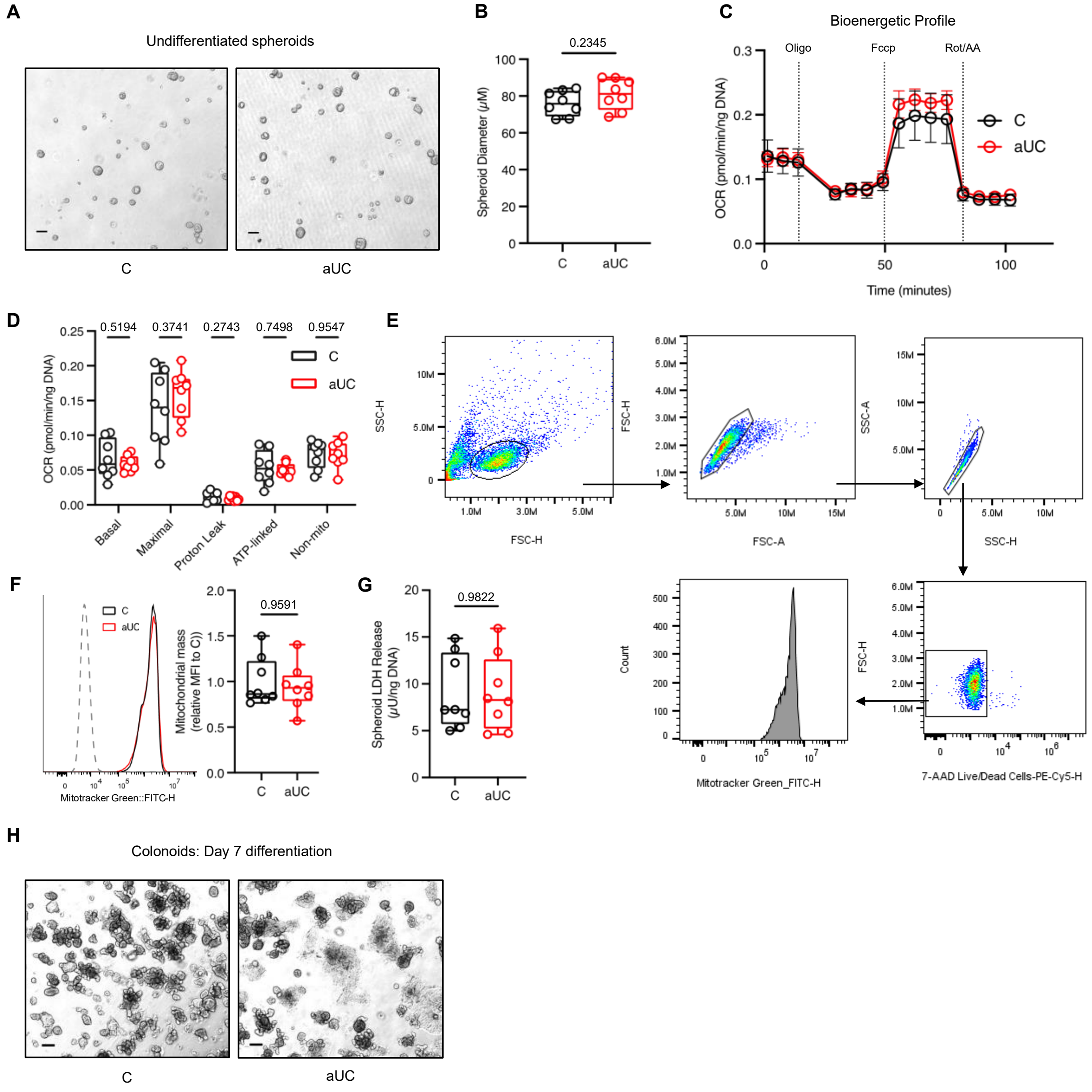


**Supplementary Fig. 2. Pediatric colon spheroids exhibit similar morphological and metabolic profiles**

(**A**) Phase contrast images of undifferentiated C and aUC spheroids after they were trypsinized, passed through a 40μm strainer and expanded in growth medium for 7 days. Scale bar, 200 μm. (**B**) The diameter of C and aUC spheroids described in **A.** Each symbol is an average measure of 20-30 spheroids of one donor. n=8 donors/group, two-sided Mann-Whitney test. (**C**) Bioenergetic profile of undifferentiated C and aUC spheroids from Seahorse MitoStress test. (**D**) OCR of C and aUC spheroids in **C**. Each symbol is an average of 4 replicates of one donor. n=8 donors/group, unpaired, two-sided t-test. (**E**) Flow cytometry gating strategy and (**F**) Estimation of mitochondrial mass with Mitotracker green intensity in undifferentiated C and aUC spheroids. Each symbol is a measure from one donor. n=8 donors/group, two-sided Mann-Whitney test. (**G**) LDH activity in C and aUC spheroids in the medium from the same plate as (**C** and **D**). n=8 donors/group, unpaired, two-sided t-test. (H) Phase contrast images of C and aUC differentiated spheroids (colonoids) on day 7 of differentiation. Scale bar, 200 μm. Where applicable, boxplots represent the first, second (median), and third quartiles with whiskers representing the minimum and maximum points. *P* values are indicated in the figures. LDH, lactate dehydrogenase; OCR, oxygen consumption rates; ODM, organoid differentiation medium; OGM, organoid growth medium

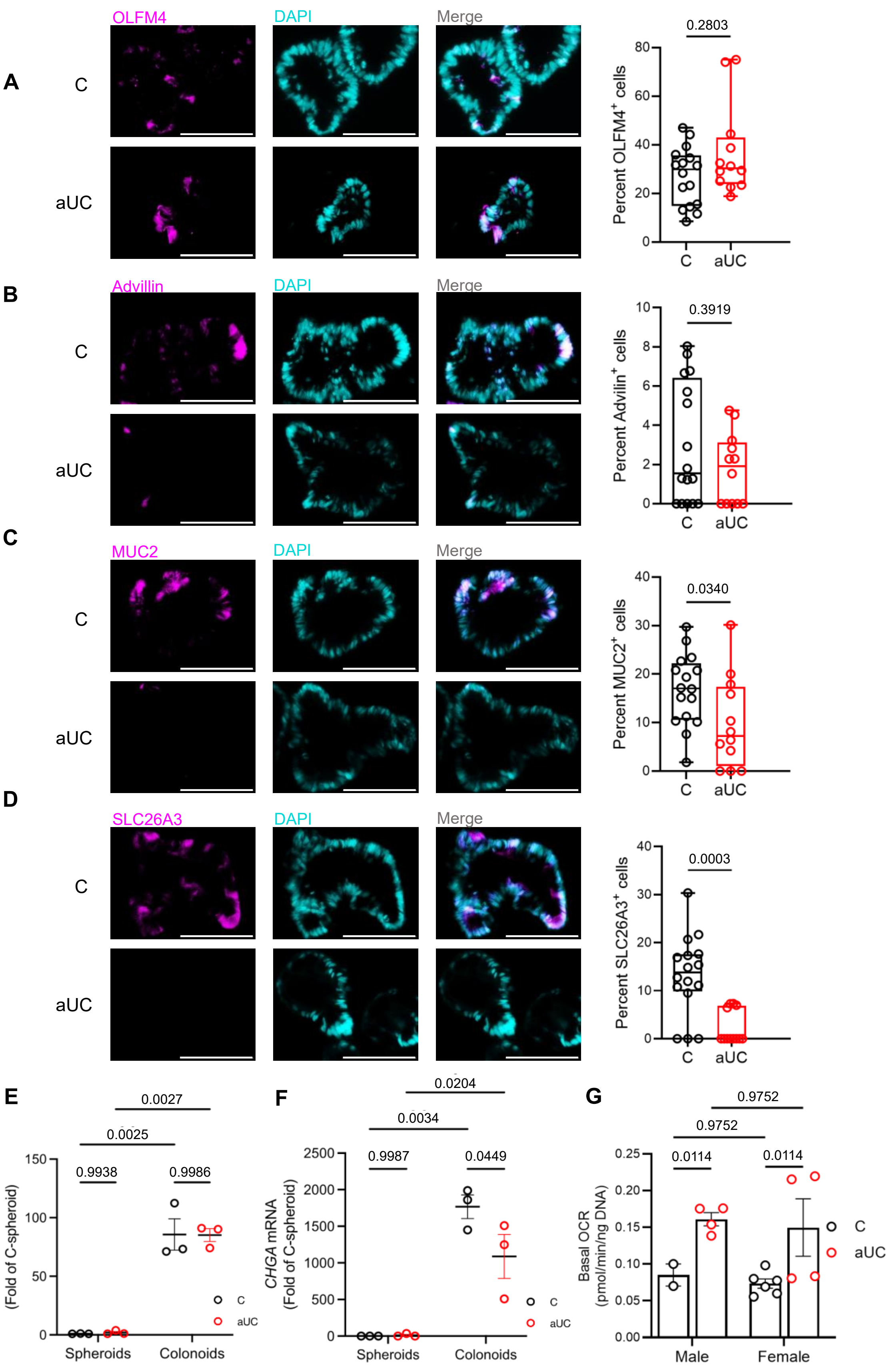
A
OLFM4
DAPI
Merge
C
aUC
0.2803
Percent OLFM4+ cells
B
Advillin
0.3919
Percent Advillin+ cells
C
MUC2
0.0340
Percent MUC2+ cells
D
SLC26A3
0.0003
Percent SLC26A3+ cells
E
ATOH1 mRNA
(Fold of C-spheroid)
0.0027
0.0025
0.9938
0.9986
Spheroids
Colonoids
F
CHGA mRNA
(Fold of C-spheroid)
0.0204
0.0034
0.9987
0.0449
G
Basal OCR
(pmol/min/ng DNA)
0.9752
0.9752
0.0114
0.0114
Male
Female

**Supplementary Fig. 3. Epithelial cell markers and metabolic response in pediatric colonoids**

(A-D) Immunofluorescent staining of OCT-embedded C and aUC colonoids after 3-day differentiation showing (A) OLFM4 (B)Advillin (C) MUC2 and (D) SLC26A3 protein expression. Each symbol represents one colonoid from 3-4 biological donors per group. Scale bar, 50 µm. Boxplot on the right represents percent of total cells within colonoid positive for protein (E-F) Spheroids were cultured in growth medium and paired samples were differentiated in differentiation medium for 3 days (colonoids) and assayed for (E) ATOH1 and (F) CHGA mRNA abundance using qRT-PCR. Each symbol represents one donor. n=3 donors/group, 2-way ANOVA with Sidak post hoc test. mean±SEM. (G) Basal OCR response of C and aUC colonoids stratified by sex, related to Fig 1F. Each symbol represents an average of 3-4 replicates for one donor. 2-way ANOVA with Sidak post hoc test. mean±SEM. Where applicable, boxplots represent the first, second (median), and third quartiles with whiskers representing the minimum and maximum points. *P* values are indicated in the figures.

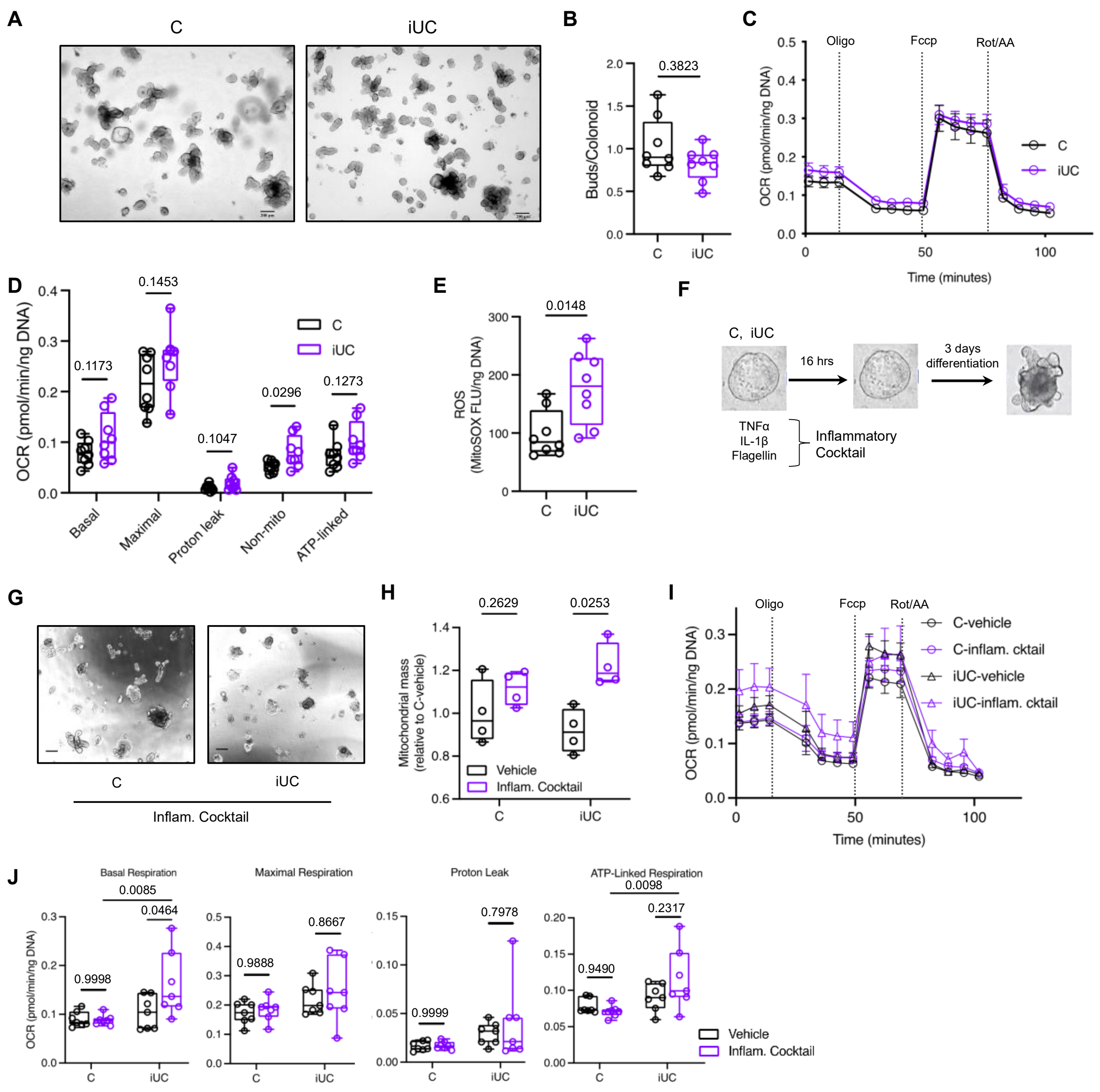


**Supplementary Fig. 4. Morphologic and Metabolic similarities in inactive UC and control colonoids**

(**A**) Phase contrast images of pediatric C and iUC colonoids after 3-day differentiation in unstimulated conditions in ODM. Scale bar, 200 µm. (**B**) The number of buds/colonoid in colonoids treated as in **A**. Each symbol is an average measure of 20-30 spheroids from one donor. n=8 donors/group, two-sided Mann-Whitney test. (**C**) Bioenergetic profile of C and iUC colonoids differentiated for 3 days as in **A**, and subjected to the Seahorse MitoStress test. n=8

donors/group. (**D**) MitoStress OCR response of C and iUC colonoids differentiated for 3 days as in **A**, in 96-well Seahorse plate. Each symbol represents an average of 3-4 replicates for one donor. n=8 donors/group, Mann-Whitney test. (**E**) The same cohort of colonoids cultured and used in **D**, were used to estimate mitochondrial ROS with the MitoSOX fluorescence assay. Each symbol represents duplicate measures for one donor. n=8 donors/group, two-sided Mann-Whitney test. (**F**) C, and iUC spheroids were exposed to an inflammatory cocktail (40 ng/mL TNFa, 20 ng/mL IL-1b, and 500 ng/mL Flagellin) in OGM for 16 hrs before differentiation in ODM for 3 days without inflammatory cocktail. (**G**) Phase contrast images of C and iUC colonoids treated as in **F**. Scale bar, 200 µm. (**H**) Estimation of mitochondrial mass with Mitotracker green intensity in C and iUC colonoids treated as in **F**. Each symbol represents one donor. n=4 donors/group, 2-way ANOVA, with Tukey correction. (**I**) Bioenergetic profile of C and iUC colonoids treated as in **F**, and subjected to the Seahorse MitoStress test. n=4 donors/group. (**J**) MitoStress OCR response of C and iUC colonoids treated as in **F**. Each symbol represents an average OCR of 3 replicates for one donor. n=7 donors/group, 2-way ANOVA with Tukey correction. Where applicable, boxplots represent the first, second (median), and third quartiles with whiskers representing the minimum and maximum points. *P* values are indicated in the figures. OCR, oxygen consumption rates; ODM, organoid differentiation medium; OGM, organoid growth medium; ROS, reactive oxygen species

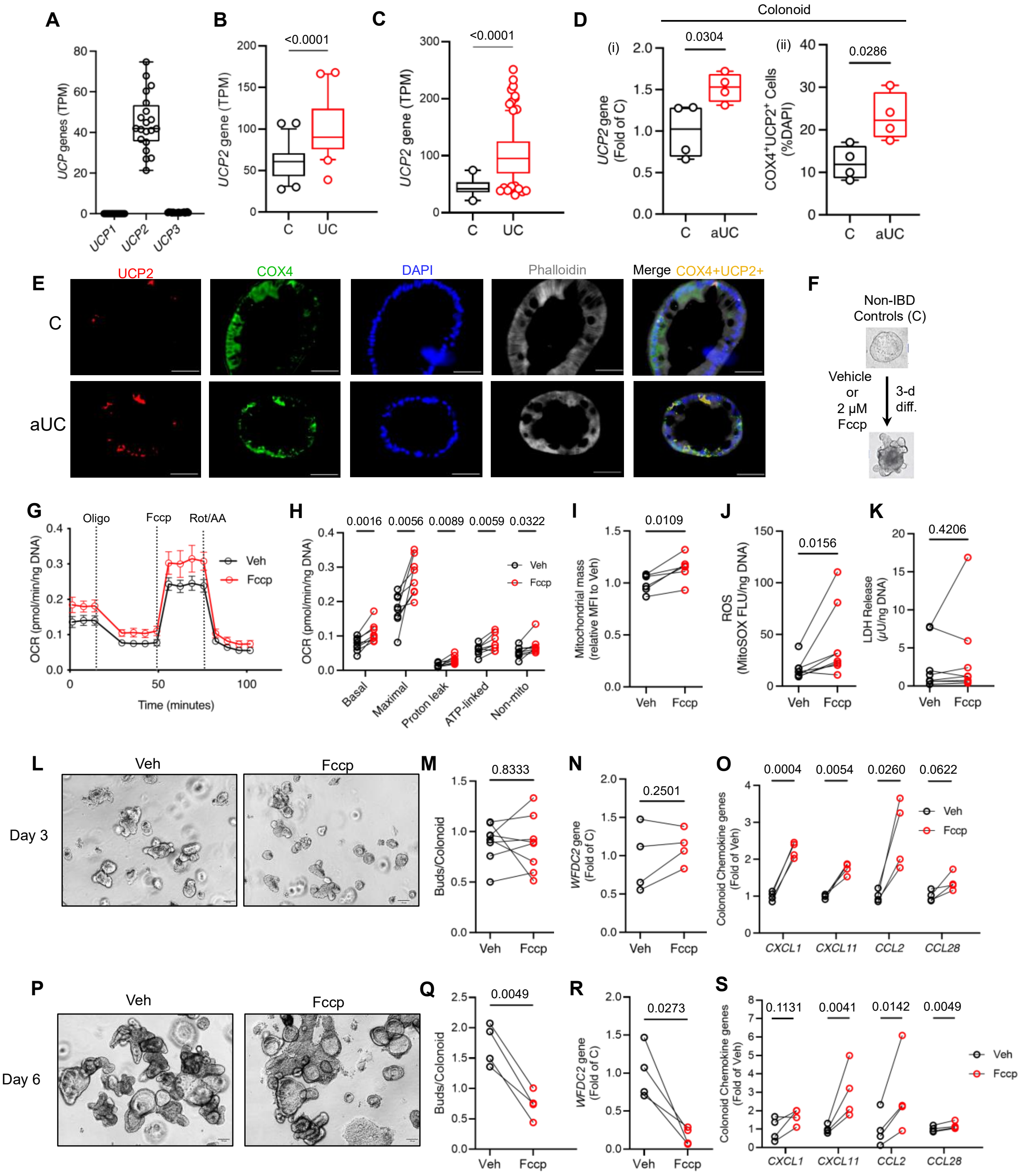

**Supplementary Fig. 5. Metabolic uncoupling contributes to hypermetabolic phenotypes during colonoid differentiation**

(**A**) Rectal gene expression of uncoupling proteins in bulk RNA-Seq data from non-IBD control participants in the NIH PROTECT study (GSE109142) showing dominant UCP2 gene expression

over other uncouplers (transcripts per million (TPM) values). n=20. (**B**) Rectal UCP2 gene expression (TPM) from treatment-naïve participants with UC in the RISK cohort (GSE117993)[1]. n=55(C) and 43(aUC) /group. Two-tailed Mann-Whitney test. (**C**) Rectal UCP2 gene expression (TPM) from the NIH PROTECT study (GSE109142)[2] of treatment-naive pediatric UC patients. n=20(C) and 206(aUC) /group. Symbols in **B** and **C** represent outliers and boxplots represent data within the interquartile range and whiskers extending to the 5th and 95th percentiles, Two-tailed Mann-Whitney test. (**D**) (i) UCP2 gene expression assessed by qRT-PCR and (ii) UCP2 protein expression in pediatric C and aUC colonoids differentiated for 3 days. Each symbol represents one donor. n=4 donors/group, (i) two-tailed unpaired t-test and (ii) two-sided Mann-Whitney test. Boxplots represent the first, second (median), and third quartiles with whiskers representing the minimum and maximum points. (**E**) C and aUC colonoids were embedded in OCT after 3-day differentiation. Representative immunofluorescence images of C and aUC colonoids after 3-day differentiation showing UCP2 expression with the mitochondrial marker, COX4. Semi-quantitation of COX4+UCP2+ cell counts is presented in **D**(ii). Scale bar, 50 µm. (**F**) Morphological and metabolic effects of mitochondrial uncoupling during colonoid differentiation were tested in C colonoids treated with or without the mitochondrial protonophore, FCCP. (**G**) Bioenergetic profile of C colonoids treated as in **F**, and subjected to the Seahorse MitoStress test. n=8 donors/group. (**H**) MitoStress OCR response of C colonoids treated as in **F**. Each symbol represents average measures of 3-4 replicates for one donor. n=8 donors/group, two-sided paired t-test. (**I**) Estimation of mitochondrial mass with Mitotracker green intensity in C colonoids treated as in **F**. Each symbol represents one donor. n=6 donors/group, two-sided paired t-test. (**J**) Mitochondrial ROS with the MitoSOX fluorescence assay in C colonoids treated as in **F**. Each symbol represents duplicate measures for one donor. n=7 donors/group, two-sided Wilcoxon

signed-rank test. (**K**) LDH activity in the medium after differentiation for 2 days. n=8 donors/group, two-sided paired t-test. (**L-S**) Control colonoids treated with vehicle (DMSO) or Fccp for 3 days (**L-O**) or 6 days (**P-S**). (**L,P**) Representative phase contrast images, Scale bar, 100 µm. (**M,Q**) The number of buds/colonoids. n=4-8 donors/group. (**N,R**) WFDC2 gene expression assessed by qRT-PCR. n=4 donors/group. (**O,S**) Gene expression of chemokines assessed by qRT-PCR. n=4 donors/group (**M-O, Q-S**) analyzed by two-sided paired t-test. Each symbol in all figures represents one donor. *P* values are indicated in the figures. LDH, lactate dehydrogenase; OCR, oxygen consumption rates; ODM, organoid differentiation medium; ROS, reactive oxygen species

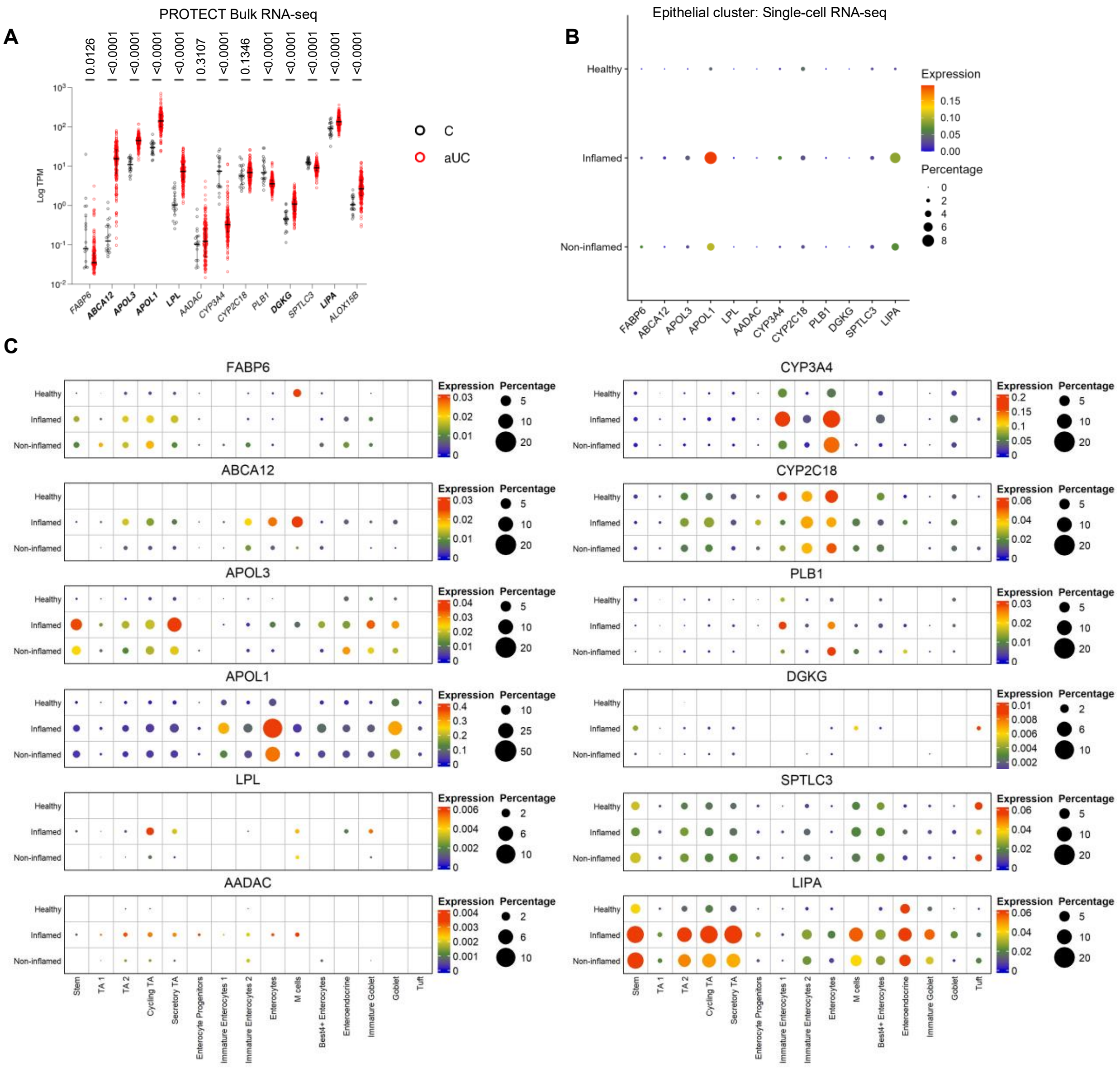


**Supplementary Fig. 6. Dysregulated lipid metabolism genes in the colon of active UC patients**

(**A**) Rectal gene expression (TPM) of lipid-related colonoid genes (as in Fig. 4**A**) from the NIH PROTECT study[2] of treatment-naive pediatric UC patients. n=20(C) -206(aUC)/group. Kruskal-Wallis test with Dunn's multiple comparisons. (**B**) Expression of colonoid lipid-related genes (as in Fig. 4A) by disease state in publicly available adult single-cell (sc) RNA-seq data[3] from donors with healthy, inflamed, and non-inflamed colon. Data represents expression from the total

epithelial cluster and is colored by relative gene expression and the relative size of each dot shows the percentage of cells expressing each marker per disease state. (**C**) Expression of colonoid lipid-related genes (as in Fig. 4A) in scRNA-seq data[2] by epithelial cluster. Data is colored by relative gene expression and the relative size of each dot shows the percentage of cells expressing each marker per epithelial cluster. *P* values are indicated in the figures.

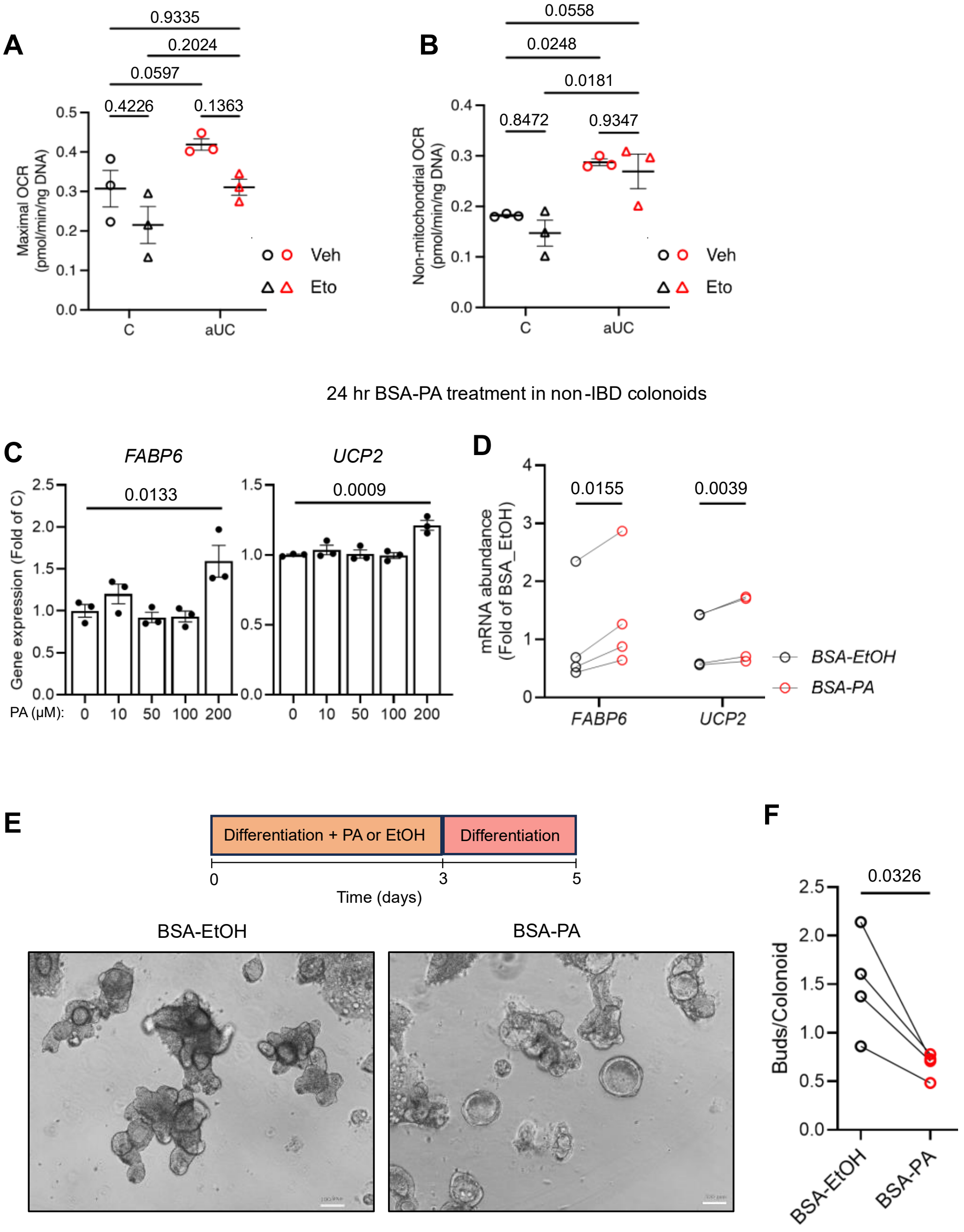

**Supplemental Fig. 7. Lipid exposure impacts epithelial colonoid metabolism and differentiation**

(**A**) Maximal OCR, and (**B**) Non-mitochondrial OCR response of C and aUC colonoids treated with or without Etomoxir. Related to **Fig. 4G-K**. n=3 donors/group. 2-way ANOVA, Tukey's *post hoc* test. mean±SEM (**C**) Dose-dependent effect of Palmitic acid on lipid transporter (*FABP6*) and uncoupling (*UCP2*) genes in non-IBD colonoids differentiated in ODM for 24 hrs and assayed by qRT-PCR. Symbols represent 3 replicates in independent matrigel domes from one donor. One-way ANOVA and Dunnett tests. mean±SEM (**D**) Colonoids from 4 non-IBD donors exposed to 200 μM BSA-PA or BSA-EtOH for 24 hours and assayed for FABP6 and UCP2 genes by qRT-PCR. Two-tailed paired t-test. (**E**) Representative phase contrast images of colonoids from 4 non-IBD donors exposed to 200 μM BSA-PA or BSA-EtOH control for the first 3 days before further differentiation up to 5 days. Scale bar, 100 μm. (**F**) The number of buds/colonoids of samples treated as in (**E**). n=4 donors/group. Two-tailed paired t-test. *P* values are indicated in the figures. BSA, bovine serum albumin; Eto, etomoxir; OCR, oxygen consumption rates; ODM, organoid differentiation medium; PA, Palmitic acid

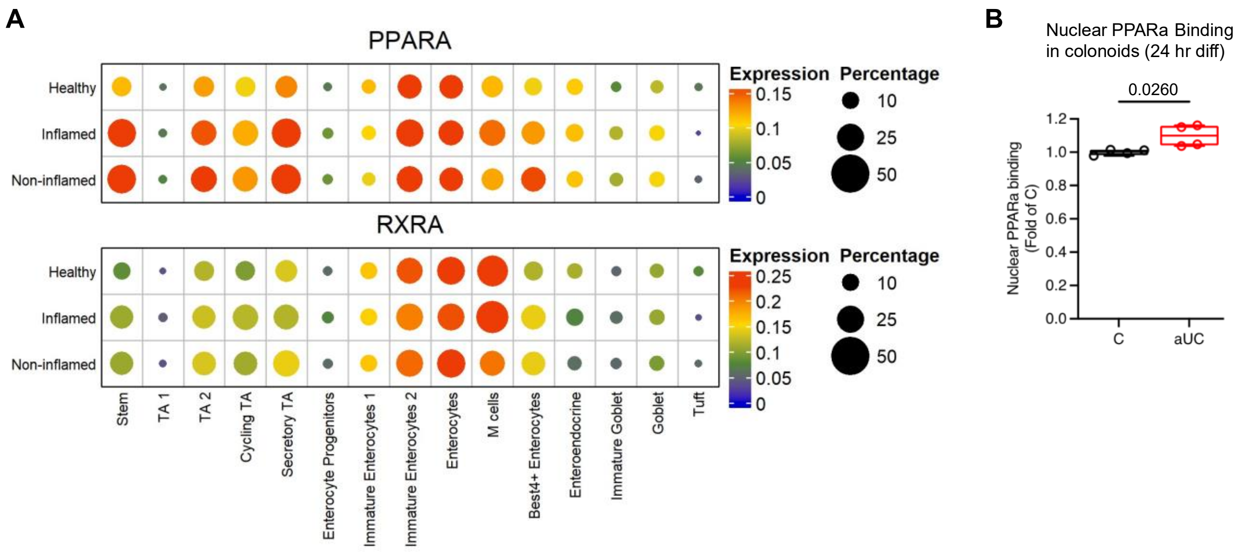


**Supplementary Fig. 8. Epithelial PPARA expression in active UC**

(**A**) Colon scRNA-seq data[3] showing the expression of PPARA and RXRA by disease state in colon epithelial lineages. Data is colored by relative gene expression and the relative size of each dot shows the percentage of cells expressing each marker per disease state. (**B**) PPAR-α activity assay in nuclear extracts of C and aUC colonoids differentiated for 24 hrs. Symbols represent four technical replicates from pooled nuclear extracts of 3 donors/group. Data is presented relative to non-IBD controls. Two-sided unpaired t-test. *P* value is indicated in the figures.

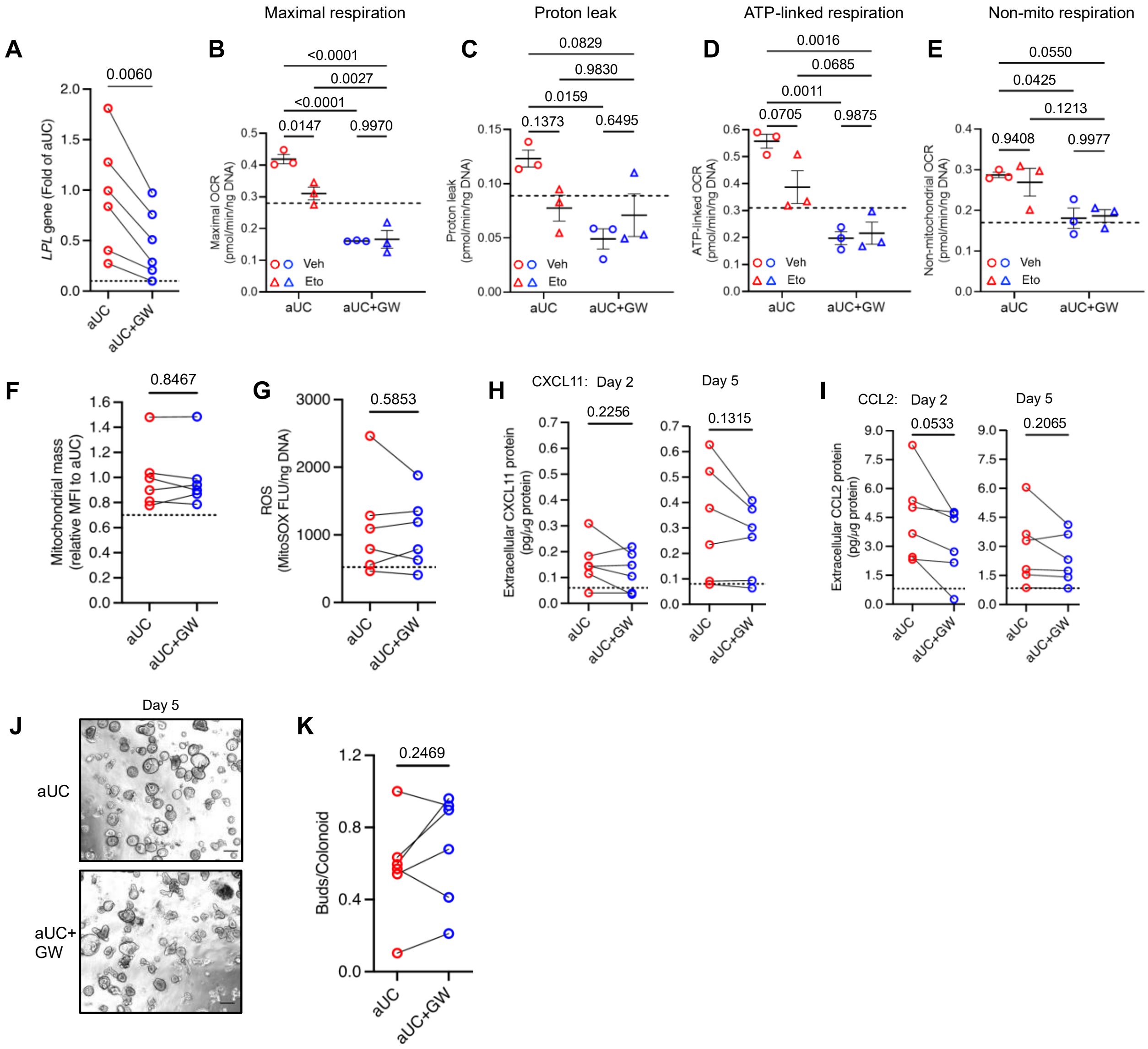


**Supplementary Fig. 9. Blockade of PPAR-α suppresses hypermetabolic features in active UC colonoids**

aUC colonoids were treated with 1 μM of the PPAR-α antagonist, GW6471 (GW), or vehicle (EtOH) during differentiation. (**A**) Effect of GW on *LPL* gene expression by qRT-PCR. Each symbol represents one donor. n=6 donors/group, two-sided paired t-test. (**B**) Maximal, (**C**) Proton leak, (**D**) ATP-linked, and (**E**) Non-mitochondrial OCR response of aUC colonoids differentiated for 3 days in ODM with or without GW and subjected to the Seahorse MitoStress test in a nutrient-deprived medium with or without Etomoxir. Each symbol represents an average

of four replicates of one donor. n=3 donors/group. 2-way ANOVA, Tukey's *post hoc* test. mean±SEM. (**F**) Estimation of mitochondrial mass with Mitotracker green intensity in aUC colonoids treated with or without GW in ODM for 3 days. Each symbol represents one donor. n=6 donors/group, two-sided Wilcoxon signed-rank test. (**G**) ROS estimate with the MitoSOX fluorescence assay in a UC colonoids differentiated for 3 days with or without GW. Each symbol represents one donor. n=6 donors/group, two-sided Wilcoxon signed-rank test. (**H)** aUC colonoids with or without GW were differentiated in ODM for 5 days. ODM collected on day 2 and on day 5 (representing the last 3 days of differentiation) was used to assess CXCL11 and (**I**) CCL2 secretion. Each symbol is an average of duplicate measures of one donor. n=6 donors/group, two-sided paired t-test. (**J**) Representative phase contrast images of aUC colonoids differentiated in ODM with or without GW for 5 days. Scale bar, 200 µm. (**K**) The number of buds/colonoid in aUC colonoids treated as in **J**. Each symbol is an average measure of 25-40 spheroids from one donor. n=6 donors/group, two-sided Wilcoxon signed-rank test. *P* values are indicated in the figures. OCR, oxygen consumption rates; ODM, organoid differentiation medium; MFI, mean fluorescence intensity

## Supplementary Tables

### Supplementary Table 1. Donor demographic and clinical information

| | *n* | Control | *n* | Inactive UC | *n* | Active UC |
|---|---|---|---|---|---|---|
| | 8 | | 8 | | 8 | |
| Age, mean ± SD | 8 | 15.4 ± 2.5 | 8 | 14.1 ± 2.4 | 8 | 13.1 ± 3.2 |
| Sex, *n* (%) | 8 | | 8 | | 8 | |
| Male | | 2 (25) | | 7 (88) | | 4 (50) |
| Female | | 6 (75) | | 1 (13) | | 4 (50) |
| Race, *n* (%) | 8 | | 8 | | 8 | |
| Black | | 2 (25) | | 0 | | 0 |
| Asian | | 0 | | 0 | | 1 (12.5) |
| White | | 6 (75) | | 8 (100) | | 7 (87.5) |
| Endoscopic Mayo Score, n (%) | 0 | | 7 | | 8 | |
| 0 (Normal) | | — | | 7 (100) | | 0 (0) |
| 1 (Mild) | | — | | 0 (0) | | 1 (12.5) |
| 2 (Moderate) | | — | | 0 (0) | | 6 (75) |
| 3 (Severe) | | — | | 0 (0) | | 1 (12.5) |
| PUCAI Score, n (%) | 0 | | 8 | | 8 | |
| 0 – 5 (Quiescent) | | — | | 8 (100) | | 2 (25) |
| 10–30 (Mild) | | — | | 0 (0) | | 3 (37.5) |
| 35–60 (Moderate) | | — | | 0 (0) | | 3 (37.5) |
| 65-85 (Severe) | | — | | 0 (0) | | 0 (0) |
| Disease Location, n (%) | 0 | | 8 | | 8 | |
| Left-sided colitis | | — | | 1 (12.5) | | 1 (12.5) |
| Extensive/Pancolitis | | — | | 7 (87.5) | | 7 (87.5) |
| Treatment, n (%) | 0 | | 8 | | 7 | |
| Oral 5-ASA | | 0 (0) | | 7 (88) | | 5 (63) |
| Oral steroids | | 0 (0) | | 0 (0) | | 3 (38) |
| Rectal steroids | | 0 (0) | | 0 (0) | | 2 (25) |
| Anti-TNF biologic | | 0 (0) | | 2 (25) | | 1 (13) |
| Vedolizumab | | 0 (0) | | 0 (0) | | 1 (13) |

**Supplementary Table 2. Taqman probes used for qPCR**

| Manufacturer | Gene name | Species | Probe ID |
|---|---|---|---|
| Invitrogen | *ACTB* | Human | Hs01060665_g1 |
| Invitrogen | *ASCL2* | Human | Hs00270888_s1 |
| Invitrogen | *ATOH1* | Human | Hs00944192_s1 |
| Invitrogen | *CCL2* | Human | Hs00234140_m1 |
| Invitrogen | *CCL28* | Human | Hs00219797_m1 |
| Invitrogen | *CHGA* | Human | HS00900370_m1 |
| Invitrogen | *CXCL1* | Human | Hs00236937_m1 |
| Invitrogen | *CXCL11* | Human | Hs00171138_m1 |
| Invitrogen | *FABP6* | Human | Hs01031183_m1 |
| Invitrogen | *GAPDH* | Human | Hs02758991_g1 |
| Invitrogen | *LGR5* | Human | Hs00969422_m1 |
| Invitrogen | *LPL* | Human | Hs00173425_m1 |
| Invitrogen | *MUC2* | Human | Hs00159374_m1 |
| Invitrogen | *UCP2* | Human | Hs01075227_m1 |
| Invitrogen | *WFDC2* | Human | Hs00196109_m1 |

## Supplementary Methods

### Lipidomics Sample Preparation

Colonoids from 3 donors per group were differentiated for 3 days and digested into single cells as previously described. Single cells were counted, and lipids were extracted in $0.5 \times 10^6$ cells using 100 ul of 1:1 butanol/methanol with 10mM ammonium formate, vortexed and bath-sonicated for 1 hr at 21-25ºC. Following sonication, samples were centrifuged (15,000x g, 10 mins, 20ºC). Subsequently, 38 uL of supernatant was transferred to a glass vial with an insert and spiked with 2 uL of Avanti's Splash II Lipidomix mass spec standard as internal standards. The mixture was vortexed for the LC/MS targeted lipidomics analysis.

### Liquid Chromatography and Triple Quadrupole Mass Spectrometry

Targeted lipidomics analysis was performed using a dynamic multiple reaction monitoring (dMRM) liquid chromatography-mass spectrometry (LC/MS) method. The analysis was conducted on an Agilent 1290 Bio LC system coupled to the 6495C triple quadrupole mass spectrometer. Separation was achieved using a ZORBAX Eclipse Plus C18 column (2.1 x 100 mm, 1.8 µm), with column temperature maintained at 45 °C. The mobile phase consisted of (A) 10 mM ammonium formate with 5 µM deactivator additive in a solvent mixture of 5:3:2 water:acetonitrile:2-propanoland (B) 10 mM ammonium formate in a solvent mixture of 1:9:90 water:acetonitrile:2-propanol. The LC flow rate is 0.4 mL/min and the chromatographic gradient started at 15%B, increased to 50%B (0-2.5 min), then ramped to 57%B (2.5-2.6 min), further increased to 70%B (2.6-9 min), then to 93% B (9-9.1 min), and further to 96%B (9.1- 11 min), finally ramped to 100% B (11-11.1 min), held at 100% B (11.1-14 min), then decreased to 15% B (14-14.2 min) and re-equilibrated at 15% B (14.2-18 min).

The 6495C triple quadrupole system was operated in fast polarity switching mode to enable simultaneous detection of both positive and negative ions within a single run. The following parameters were applied: capillary voltages set to +3500 V for positive ion mode and -3000V for negative ion mode; drying gas temperature at 150°C with a flow rate of 17 L/min; sheath gas temperature at 200°C with a flow rate of 10 L/min; and the delta Electron Multiplier Voltage (EMV) set to 200 V for both positive and negative ion modes. A total of 667 MRMs transitions were monitored (Positive: 648, negative: 19) with a cycle time of 750 ms.

**Lipid Quantitative Data Analysis and Statistical Analyses**

The dMRM LC/MS raw data were processed using Agilent MassHunter Quantitative Analysis Software (Version12.1). A predefined dMRM method, including all MRM transitions and retention times (RTs) for 762 targeted lipids across 44 lipid classes and 14 internal standards, was used for peak extractions. Each lipid peak integration was manually inspected to ensure accuracy and consistency. A total of 739 lipids were detected from the biological cell extracts. Quantification results were exported from the Quantitative software and converted to a generic Excel format containing lipid species name, mass, retention time, and peak area. The formatted data was then imported into Agilent Mass Profiler Professional (MPP) software (version 15.1) for statistical analysis. For the Volcano plot, we applied a moderate t-test with BenJamini Hochberg false discovery rate (FDR) for multiple testing correction. Statistically significant lipids were identified using a corrected P-value $\leq 0.05$ and a fold change $\geq 2$ as cut-off criteria when comparing the aUC vs Control.

## Supplementary References


1. Pelia, R. *et al.* Profiling non-coding RNA levels with clinical classifiers in pediatric Crohn's disease. *BMC Med. Genomics* 14, 194 (2021).

2. Haberman, Y. *et al.* Ulcerative colitis mucosal transcriptomes reveal mitochondriopathy and personalized mechanisms underlying disease severity and treatment response. *Nat. Commun.* **10**, 38 (2019).

3. Smillie, C. S. *et al.* Intra- and Inter-cellular Rewiring of the Human Colon during Ulcerative Colitis. *Cell* **178**, 714-730.e22 (2019)
