# Supplementary material for: Patient-derived colon epithelial organoids reveal lipid-related metabolic dysfunction in pediatric ulcerative colitis
Source: Nat Commun. 2025 Dec 10;16:11026. doi: 10.1038/s41467-025-65988-2 (PMC12695892; doi:10.1038/s41467-025-65988-2)
Supplement: Supplementary file 2 — Description of Additonal Supplementary Files [file 41467_2025_65988_MOESM2_ESM.pdf]

Description of Additional Supplementary Files

Supplementary Data 1
Bulk RNA-seq differentially expressed genes in undifferentiated (spheroids) and 3-day differentiated (colonoids) organoids from patients with active or inactive ulcerative colitis and non-IBD controls.

Supplementary Data 2
Molecular and Cellular Functions of colonoid Bulk RNA-seq DEGs from Ingenuity Pathway Analysis.

Supplementary Data 3
Raw lipidomics data and analyzed data in colonoids from active UC patients and non-IBD controls.

Supplementary Data 4
Causal Network analysis of colonoid Bulk RNA-seq DEGs from Ingenuity Pathway Analysis.
