# Supplementary material for: Patient-derived colon epithelial organoids reveal lipid-related metabolic dysfunction in pediatric ulcerative colitis
Source: Nat Commun. 2025 Dec 10;16:11026. doi: 10.1038/s41467-025-65988-2 (PMC12695892; doi:10.1038/s41467-025-65988-2)
Supplement: Supplementary file 7 — Reporting Summary [file 41467_2025_65988_MOESM7_ESM.pdf]

nature portfolio

Corresponding author(s): Michael J. Rosen

Last updated by author(s): Sep 22, 2025

Reporting Summary

Nature Portfolio wishes to improve the reproducibility of the work that we publish. This form provides structure for consistency and transparency in reporting. For further information on Nature Portfolio policies, see our Editorial Policies and the Editorial Policy Checklist.

Statistics

For all statistical analyses, confirm that the following items are present in the figure legend, table legend, main text, or Methods section.

n/a	Confirmed	
☐	☒	The exact sample size (*n*) for each experimental group/condition, given as a discrete number and unit of measurement
☐	☒	A statement on whether measurements were taken from distinct samples or whether the same sample was measured repeatedly
☐	☒	The statistical test(s) used AND whether they are one- or two-sided<br>*Only common tests should be described solely by name; describe more complex techniques in the Methods section.*
☒	☐	A description of all covariates tested
☐	☒	A description of any assumptions or corrections, such as tests of normality and adjustment for multiple comparisons
☐	☒	A full description of the statistical parameters including central tendency (e.g. means) or other basic estimates (e.g. regression coefficient) AND variation (e.g. standard deviation) or associated estimates of uncertainty (e.g. confidence intervals)
☐	☒	For null hypothesis testing, the test statistic (e.g. *F*, *t*, *r*) with confidence intervals, effect sizes, degrees of freedom and *P* value noted<br>*Give P values as exact values whenever suitable.*
☒	☐	For Bayesian analysis, information on the choice of priors and Markov chain Monte Carlo settings
☒	☐	For hierarchical and complex designs, identification of the appropriate level for tests and full reporting of outcomes
☒	☐	Estimates of effect sizes (e.g. Cohen's *d*, Pearson's *r*), indicating how they were calculated

Our web collection on statistics for biologists contains articles on many of the points above.

Software and code

Policy information about availability of computer code

Data collection	NovoExpress software, Mircosoft Excel, Keyence BZ-X800 software analyzer, Leica LAS X software, QuantStudio 3 thermocycler, MassHunter Quant software , SoftMax Pro software, Agilent Seahorse Wave desktop v 2.6.3
Data analysis	Graphpad Prism v.10.0, Agilent Seahorse Wave desktop v 2.6.3 , FlowJo software v.10, nf-core/rnaseq (v3.5) , Ingenuity Pathway Analysis, Agilent Mass Profiler Professional (v.15.1).

For manuscripts utilizing custom algorithms or software that are central to the research but not yet described in published literature, software must be made available to editors and reviewers. We strongly encourage code deposition in a community repository (e.g. GitHub). See the Nature Portfolio guidelines for submitting code & software for further information.

Data

Policy information about availability of data

All manuscripts must include a data availability statement. This statement should provide the following information, where applicable:

- Accession codes, unique identifiers, or web links for publicly available datasets
- A description of any restrictions on data availability
- For clinical datasets or third party data, please ensure that the statement adheres to our policy

The bulk RNA sequencing data in this study have been deposited into Gene Expression Omnibus (GEO) under the accession number GSE276170. This study used the publicly available bulk RNA sequencing datasets under the accession numbers GSE109142 and GSE117993. This study also utilized publicly available single cell RNA

sequencing data from the Single Cell Portal under the accession number SCP259. Lipidomics data are provided in Supplementary Information. Source data are provided with this paper.

Research involving human participants, their data, or biological material

Policy information about studies with human participants or human data. See also policy information about sex, gender (identity/presentation), and sexual orientation and race, ethnicity and racism.

Reporting on sex and gender	Male and female samples are represented in all group, with uneven distribution of the sex in the groups. Sex and gender were not considered for sample collected and in the design of the study.
Reporting on race, ethnicity, or other socially relevant groupings	N/A
Population characteristics	Colon organoids were generated from biosamples obtained from 24 participants. Donors have an average age of 14.2 years, with 54% male.
Recruitment	Participants were recruited following their clinically scheduled endoscopy. Consent was obtained from parents or legal guardians and assent was obtained from each patient donor.
Ethics oversight	Institutional Review Boards at Cincinnati Children's Hospital Medical Center, Cincinnati, Ohio, and Lucile Packard Children's Hospital Stanford, Palo Alto, California.

Note that full information on the approval of the study protocol must also be provided in the manuscript.

Field-specific reporting

Please select the one below that is the best fit for your research. If you are not sure, read the appropriate sections before making your selection.

☒ Life sciences ☐ Behavioural & social sciences ☐ Ecological, evolutionary & environmental sciences

For a reference copy of the document with all sections, see nature.com/documents/nr-reporting-summary-flat.pdf

Life sciences study design

All studies must disclose on these points even when the disclosure is negative.

Sample size	For experiments comparing metabolic measures between diagnoses, a sample size of 8 patient colonoid lines per group was chosen to provide 80% power to detect a difference of 1.5-1.8 times the standard deviation between groups. Based on initial results from metabolic studies and RNA-seq, and with inhibitor studies, larger effect sizes were anticipated and a minimum n of 3 per group were used.
Data exclusions	Analyses were performed on all experimental data.
Replication	All data used biological replicates and most individual assays were conducted with at least 2 technical replicates for each biological donor.
Randomization	No randomization was done. Samples were assigned for experimental use based on the clinical diagnosis of the donor.
Blinding	All measurements of spheroid diameter, colonoid budding and immunofluorescence analyses were performed by an investigator blinded to the diagnosis groups. Investigators were not blinded for other experiments.

Reporting for specific materials, systems and methods

We require information from authors about some types of materials, experimental systems and methods used in many studies. Here, indicate whether each material, system or method listed is relevant to your study. If you are not sure if a list item applies to your research, read the appropriate section before selecting a response.

Materials & experimental systems

n/a	Involved in the study
☐	☒ Antibodies
☐	☐ Eukaryotic cell lines
☐	☐ Palaeontology and archaeology
☐	☐ Animals and other organisms
☐	☐ Clinical data
☐	☐ Dual use research of concern
☐	☐ Plants

Methods

n/a	Involved in the study
☒	☐ ChIP-seq
☐	☒ Flow cytometry
☒	☐ MRI-based neuroimaging

Antibodies

Antibodies used

UCP2 (1:100, Proteintech, #11081-1-AP)
COX4 (1:200, Invitrogen, #GT6310)
AVIL (1:100, Sigma-Aldrich, #HPA058864)
OLFM4 (1:140, Abcam, #ab85046)
SLC26A3 (1:100, Novus Biologicals, NBP1-84450)
MUC2 (1:200, Invitrogen, #MA5-12345)
Alexa Fluor 647 (1;1000, Invitrogen, #A31573)
Alexa Fluor 488 (1:1000, Invitrogen, #A11001)
Alexa Fluor 594 (1:100, Invitrogen, #A37117)
Alexa Fluor 488 (1:400, Invitrogen, #A32766)

Validation

All the antibody mentioned above was validated for usage of each species based on the manufacture's website of each product.

Eukaryotic cell lines

Policy information about cell lines and Sex and Gender in Research

Cell line source(s)

Colon organoids derived from primary colon epithelial crypts. Details provided in methods.

Authentication

n/a

Mycoplasma contamination

None

Commonly misidentified lines
(See ICLAC register)

None

Palaeontology and Archaeology

Specimen provenance

Provide provenance information for specimens and describe permits that were obtained for the work (including the name of the issuing authority, the date of issue, and any identifying information). Permits should encompass collection and, where applicable, export.

Specimen deposition

Indicate where the specimens have been deposited to permit free access by other researchers.

Dating methods

If new dates are provided, describe how they were obtained (e.g. collection, storage, sample pretreatment and measurement), where they were obtained (i.e. lab name), the calibration program and the protocol for quality assurance OR state that no new dates are provided.

☐ Tick this box to confirm that the raw and calibrated dates are available in the paper or in Supplementary Information.

Ethics oversight

Identify the organization(s) that approved or provided guidance on the study protocol, OR state that no ethical approval or guidance was required and explain why not.

Note that full information on the approval of the study protocol must also be provided in the manuscript.

Animals and other research organisms

Policy information about studies involving animals; ARRIVE guidelines recommended for reporting animal research, and Sex and Gender in Research

Laboratory animals

N/A

Wild animals

N/A

Reporting on sex	N/A
Field-collected samples	N/A
Ethics oversight	N/A

Note that full information on the approval of the study protocol must also be provided in the manuscript.

Clinical data

Policy information about clinical studies

All manuscripts should comply with the ICMJE guidelines for publication of clinical research and a completed CONSORT checklist must be included with all submissions.

Clinical trial registration	N/A
Study protocol	N/A
Data collection	N/A
Outcomes	N/A

Dual use research of concern

Policy information about dual use research of concern

Hazards

Could the accidental, deliberate or reckless misuse of agents or technologies generated in the work, or the application of information presented in the manuscript, pose a threat to:

No	Yes	
☒	☐	Public health
☒	☐	National security
☒	☐	Crops and/or livestock
☒	☐	Ecosystems
☒	☐	Any other significant area

Experiments of concern

Does the work involve any of these experiments of concern:

No	Yes	
☒	☐	Demonstrate how to render a vaccine ineffective
☒	☐	Confer resistance to therapeutically useful antibiotics or antiviral agents
☒	☐	Enhance the virulence of a pathogen or render a nonpathogen virulent
☒	☐	Increase transmissibility of a pathogen
☒	☐	Alter the host range of a pathogen
☒	☐	Enable evasion of diagnostic/detection modalities
☒	☐	Enable the weaponization of a biological agent or toxin
☒	☐	Any other potentially harmful combination of experiments and agents

Plants

Seed stocks | N/A

Novel plant genotypes | N/A

Authentication | N/A

Flow Cytometry

Plots

Confirm that:

☒ The axis labels state the marker and fluorochrome used (e.g. CD4-FITC).

☒ The axis scales are clearly visible. Include numbers along axes only for bottom left plot of group (a 'group' is an analysis of identical markers).

☒ All plots are contour plots with outliers or pseudocolor plots.

☐ A numerical value for number of cells or percentage (with statistics) is provided.

Methodology

Sample preparation

Single cells were released from organoids by the addition of 900 µL TrypLE Express followed by incubation at 37oC for 10 mins vortexing for 20 seconds with 10-second intervals. Pellets were and resuspended in 150 nM mtGreen solution prepared in phenol-red free DMEM/F12 + 5% FBS, followed by incubation for 30 mins at 37oC in a 5% CO2 incubator. Cells were washed twice in PBS and resuspended in 500 µL of ice-cold PBS. The cells were passed through Falcon FACS tubes with a cell strainer cap and incubated with 5 µL of the live-dead stain, 7-Aminoactinomycin D (7-AAD), for 5 mins on ice.

Instrument

Agilent NovoCyte Flow Cytometer

Software

The NovoExpress software (v. 1.6.2)
Flowjo (v. 10) was used for analyses.
Prism (v.10.0)
Agilent MassHunter Quantitative Analysis Software (Version12.1).
Agilent Mass Profiler Professional (MPP) software (version 15.1)
Keyence BZ-X800 software (v. 1.1.1.8)
Leica LAS X software (v. 5.0.2)
R (v.4.3.3) package Seurat (v.5.1.0).

Cell population abundance

The 7-AAD negative population was abundant

Gating strategy

The mean fluorescence intensity (MFI) of mtGreen was measured using the FITC channel and following 7-AAD exclusion (PE-Cy5).

☒ Tick this box to confirm that a figure exemplifying the gating strategy is provided in the Supplementary Information.
